# (2*R*,5*S*)‐Theaspirane Identified as the Kairomone for the Banana Weevil, *Cosmopolites sordidus,* from Attractive Senesced Leaves of the Host Banana, *Musa spp*.

**DOI:** 10.1002/chem.201800315

**Published:** 2018-06-06

**Authors:** Samson A. Abagale, Christine M. Woodcock, Antony M. Hooper, John C. Caulfield, David Withall, Keith Chamberlain, Samuel O. Acquaah, Helmut Van Emden, Haruna Braimah, John A. Pickett, Michael A. Birkett

**Affiliations:** ^1^ Crops Research Institute Council for Scientific and Industrial Research P.O. Box 3785 Fumesua-Kumasi Ghana; ^2^ Department of Chemistry Kwame Nkrumah University of, Science and Technology, PMB Kumasi Ghana; ^3^ Department of Biointeractions and Crop Protection, Rothamsted Research Harpenden Hertfordshire AL5 2JQ UK; ^4^ School of Biological and Chemical Sciences Queen Mary University of London London E1 4NS UK; ^5^ School of Agriculture, Policy and Development The University of Reading, Earley Gate P.O. Box 237 Reading Berkshire RG6 6AR UK; ^6^ School of Chemistry Cardiff University Cardiff Wales CF10 3AT UK

**Keywords:** banana weevil, chiral GC, electrophysiology, kairomone, mass spectrometry

## Abstract

The principal active component produced by highly attractive senesced host banana leaves, *Musa* spp., for the banana weevil, *Cosmopolites sordidus*, is shown by coupled gas chromatography‐electroantennography (GC‐EAG), coupled GC‐mass spectrometry (GC‐MS), chemical synthesis and coupled enantioselective (chiral) GC‐EAG to be (2*R*,5*S*)‐theaspirane. In laboratory behaviour tests, the synthetic compound is as attractive as natural host leaf material and presents a new opportunity for pest control.

The banana weevil, *Cosmopolites sordidus* Germar (Coleoptera, Curculionidae), is the most important insect pest of bananas and plantains, *Musa spp*.^1^, ^2^, ^3^ throughout the world. Feeding damage is caused by larvae of *C. sordidus* which are protected within the plant tissue, and so management strategies target adult weevils. Pheromones and other semiochemicals (naturally occurring behaviour‐ or development‐modifying chemicals) constitute important tools for monitoring and detecting insect populations. A male‐produced aggregation pheromone, (1*S*,3*R*,5*R*,7*S*)‐sordidin, has been identified for *C. sordidus*.^4^ For smallholder farmers in Ghana, for whom banana and plantain provide staple food, (1*S*,3*R*,5*R*,7*S*)‐sordidin is deemed to be too expensive, and alternative semiochemical‐based tools are urgently sought. Previous studies have shown that host plant location by adult *C. sordidus* is influenced by a highly attractive volatile kairomone from senesced banana leaves,^5^, ^6^ which, if identified, could provide an effective and affordable alternative lure for management of *C. sordidus* on smallholder farms. The purpose of this work was to identify the active component(s) from volatile material collected from senesced leaves, using coupled gas chromatography‐electroantennography (GC‐EAG) recordings from the antennae of adult female *C. sordidus*, and confirm the attractiveness of the identified compound(s), thereby providing the quality assurance for using senesced banana leaves as an ethnobotanically based locally produced material in *C. sordidus* management.

Coupled GC‐EAG analysis (see the Supporting Information) with natural volatile material collected from senesced banana leaf material confirmed that the attractiveness of the material was caused by a very minor component with highly significant EAG activity (Figure 1). The 70 eV EI mass spectrum of the unknown EAG‐active component (Figure 2) showed a base peak at *m*/*z* 138, an additional diagnostic fragment at *m*/*z* 179 and a molecular ion at *m*/*z* 194. Comparison of this spectrum with the literature^7^, ^8^ suggested a theaspirane isomer **1**, the base peak being rationalised by loss formally of isobutene (C_4_H_8_) via a retro Diels–Alder rearrangement (Figure 2 inset). The presence of two stereocentres (at the 2‐ and 5‐positions) gives four possible stereoisomers, produced initially as the mixture, by chemoenzymatic synthesis from dihydro‐β‐ionone **2** (Scheme 1). To approach resolution of the natural EAG active isomer, initial reduction of **2** with sodium borohydride in a non‐stereospecific manner gave a mixture of the (*R*) and (*S*)‐isomers of dihydro‐β‐ionol in overall 100 % yield. The mixture of ionol isomers was resolved chemoenzymatically using lipase‐mediated acetylation (*Pseudomonas cepaciae* lipase Amano PS‐C, vinyl acetate, 99.2 % *ee R*, 94.8 % *ee S*). By adjusting incubation time, it was possible to obtain 99.1 % *ee S*. Following separation of the (*R*)‐ionol acetate and the (*S*)‐ionol by silica gel liquid chromatography, the ionol then underwent intramolecular 5‐*exo*‐trig cyclisation upon heat treatment with selenium dioxide in dioxane to generate a diastereomeric pair of theaspirane isomers ((2*S*,5*S*)‐**1**, (2*S*,5*R*)‐**1**) (see the Supporting Information), overall 35 % yield over 2 steps). Cleavage of the (*R*)‐acetate (using potassium hydroxide in aqueous methanol) followed by similar treatment of the (*R*)‐ionol with selenium dioxide in dioxane furnished the other diastereomeric pair of theaspirane isomers ((2*R*,5*R*)‐**1**, (2*R*,5*S*)‐**1**) (see the Supporting Information) in overall 41 % yield over 2 steps. The diastereoisomers were difficult to separate on silica gel (4 % diethyl ether in petroleum ether) due to their lack of polarity and so the isolated diastereomeric excesses were variable and mixed fractions reduced recovery. However, a purified enantiomer of the synthetic natural product, (2*R*,5*S*)‐**1**, was obtained in 98.7 % *ee*, 99.5 % de. To verify the relative stereochemistry, nuclear Overhauser experiments on the (2*R*,5*S*)**‐1** showed a nOe correlation between the 6‐Me groups and the H‐2 proton showing this proton must be on the face of the tetrahydrofuran moiety facing to the C‐6 gem‐dimethyl group (see the Supporting Information). Complementary verification was observed by analysing (5*R*,2*R*)**‐1** in which a nOe correlation was observed between the 2‐Me group and the C‐6 gem‐dimethyl group. Coupled enantioselective (chiral) GC‐EAG analysis (see the Supporting Information) using a mix of all four synthetic isomers revealed the relative GC retention times of the isomers (Figure 3, upper trace), and comparison with coupled enantioselective GC‐EAG analysis using the natural volatile material collected from senesced banana leaf material revealed matching GC retention times for the (2*R*,5*S*)‐isomer and the natural theaspirane isomer (Figure 3 lower trace), thus confirming the identity of the electrophysiologically active naturally occurring isomer to be (2*R*,5*S*)‐**1**.

**Figure 1:**
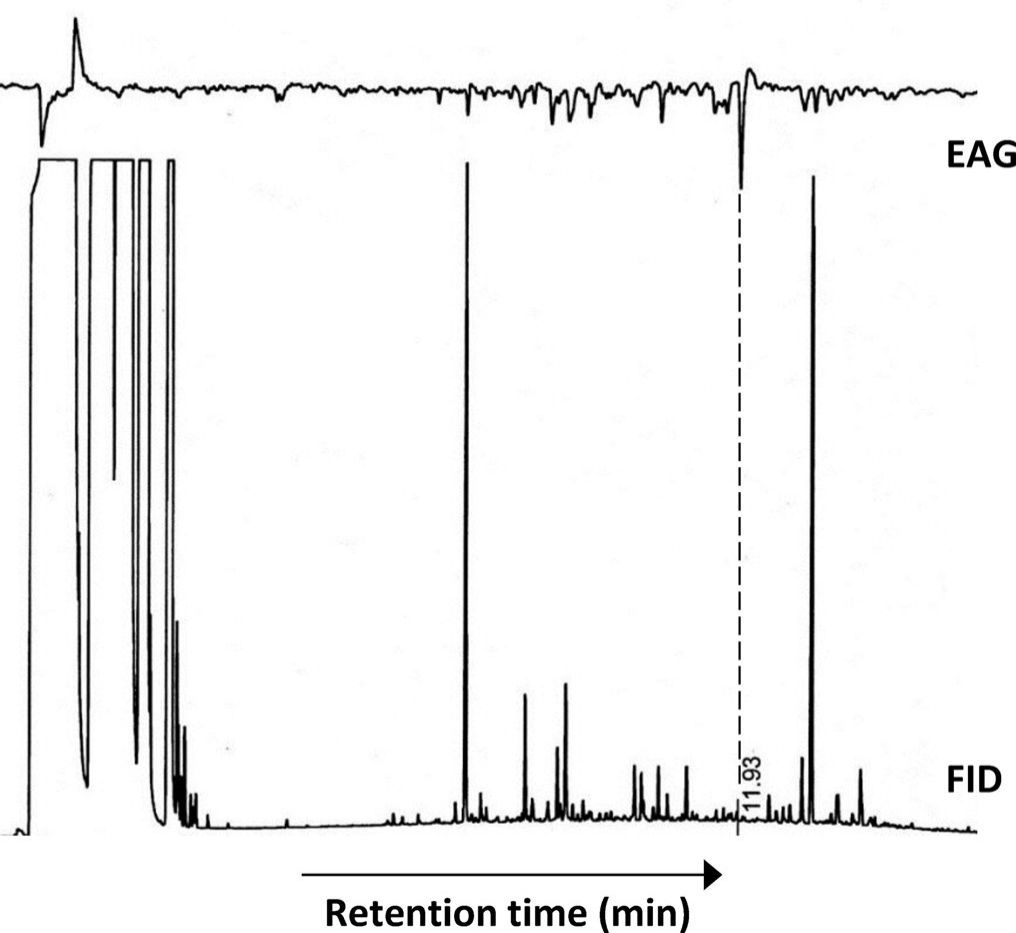
Coupled GC‐EAG responses of adult *C. sordidus* to natural volatile material collected from senesced banana leaves volatile material collected by headspace collection, on a non‐polar DB‐1 GC column. The annotated peak is a minor component with major consistent EAG activity.

**Figure 2:**
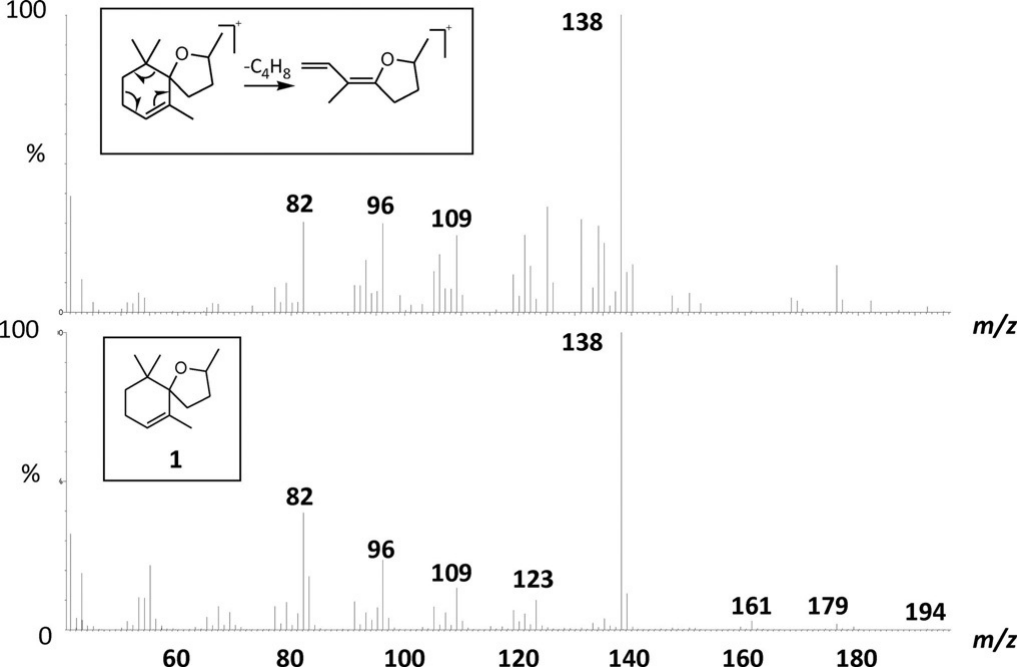
70 eV EI mass spectrum of EAG‐active compound identified from natural volatile material collected from senesced banana leaves (upper), identified as a theaspirane isomer **1** and NIST‐MS of theaspirane (lower). Inset: retro‐Diels–Alder rearrangement of parent ion from **1**.

**Scheme 1:**
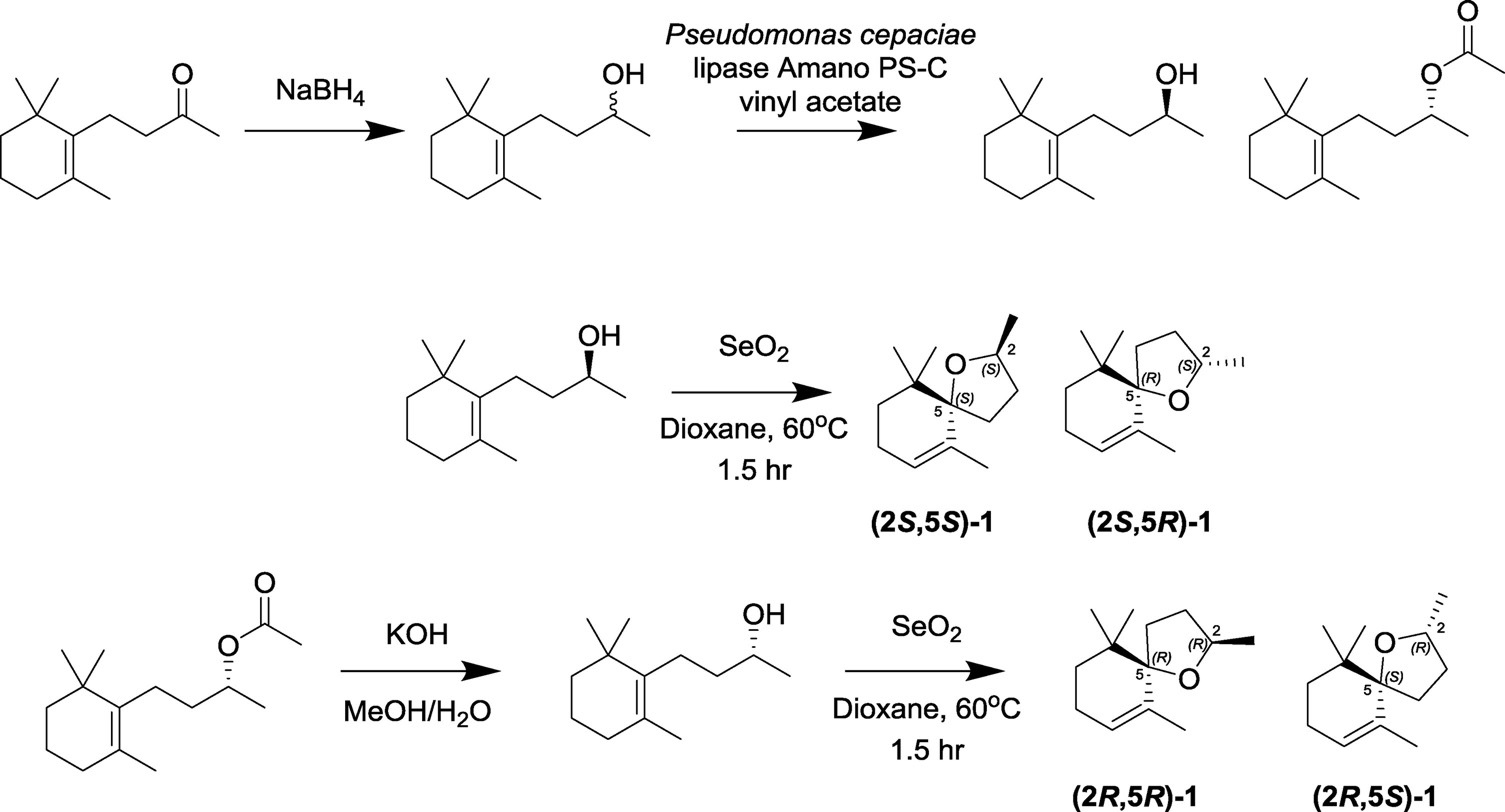
Chemoenzymatic synthesis of theaspirane isomers.

**Figure 3:**
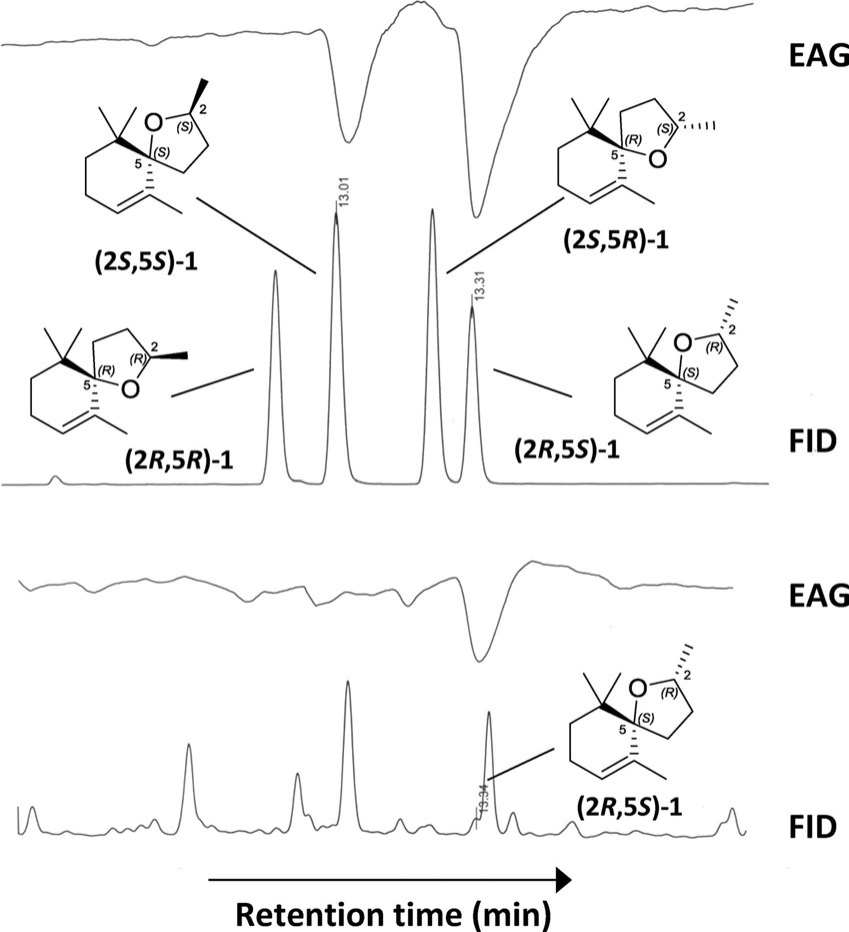
Enantioselective (chiral) coupled gas chromatography‐electroantennography (GC‐EAG) analysis of the four synthesized theaspirane isomers (upper traces) and natural volatile material collected from senesced banana leaves (lower traces), showing alignment of the (2*R*,5*S*)‐isomer **1** with the natural theaspirane isomer and the single EAG peak for the natural isomer.

In behaviour assays with female *C. sordidus* conducted in a linear three chamber olfactometer (see the Supporting Information), senesced banana leaf material and collected volatile organic compounds (VOCs) were significantly more attractive (*P*=0.013 and 0.001 respectively) than controls and were equally attractive in dual‐choice assays. A mixture of the natural (2*R*,5*S*)**‐1** and non‐natural (2*S*,5*R*)**‐1** isomers was behaviourally active at a dose of 0.2 μg and 0.02 μg (Students’ *t*‐test; *P*<0.003, *P*<0.01 respectively). A mixture of the non‐natural (2*S*,5*S*)**‐1** and (2*R*,5*R*)**‐1** isomers was shown to have behavioural activity only at a dose of 0.2 μg (*P*=0.04), in spite of the observed EAG activity for (2*S*,5*S*)**‐1**. A mixture of all four isomers of **1** was behaviourally active at all doses tested, that is, 2 (tested twice), 0.2 and 0.02 μg (*P*=0.001, 0.017, 0.001 and 0.002, respectively). When tested in combination with commercially available sordidin (Cosmolure), a mixture of (2*R*,5*S*)**‐1** and (2*S*,5*R*)**‐1** at a dose of 0.05 μg synergised the activity of the pheromone (*P*=0.04). The EAG data suggests that antennal detection of the theaspiranes requires a particular structural motif, that is, 5*S* stereochemistry, but that a specific overall 3D structure of the compound (2*R*,5*S*), is required to elicit the behavioural response in adult female *C. sordidus*. Our data suggest that the newly identified compound (2*R*,5*S*)**‐1**, present in minor quantities in senesced banana leaf material, is responsible for the attraction of adult female *C. sordidus* and is therefore the major kairomone component. The identification provides the quality assurance for the deployment of readily available senesced banana leaf material, or locally produced extracts thereof, as a lure component of affordable trapping technology that can manage *C. sordidus* on smallholder banana and plantain farms.

## Conflict of interest

The authors declare no conflict of interest.

## Supporting information

As a service to our authors and readers, this journal provides supporting information supplied by the authors. Such materials are peer reviewed and may be re‐organized for online delivery, but are not copy‐edited or typeset. Technical support issues arising from supporting information (other than missing files) should be addressed to the authors.

Supplementary
