# Supplementary material for: (2R,5S)‐Theaspirane Identified as the Kairomone for the Banana Weevil, Cosmopolites sordidus, from Attractive Senesced Leaves of the Host Banana, Musa spp
Source: Chemistry. 2018 Jun 6;24(37):9217–9. doi: 10.1002/chem.201800315 (PMC6055713; doi:10.1002/chem.201800315)
Supplement: Supplementary file 1 — Supplementary [file CHEM-24-9217-s001.pdf]

CHEMISTRY
A European Journal

Supporting Information

(2*R*,5*S*)-Theaspirane Identified as the Kairomone for the Banana Weevil, *Cosmopolites sordidus*, from Attractive Senesced Leaves of the Host Banana, *Musa spp.*

Samson A. Abagale,[a, b] Christine M. Woodcock,[c] Antony M. Hooper,[d] John C. Caulfield,[c] David Withall,[c] Keith Chamberlain,[c] Samuel O. Acquaah,[b] Helmut Van Emden,[e] Haruna Braimah,*[a] John A. Pickett,[f] and Michael A. Birkett*[c]

chem_201800315_sm_miscellaneous_information.pdf

Supporting Information

General information

All chemicals were purchased from Sigma-Aldrich unless otherwise stated. All were of analytical quality or better and used as received unless otherwise stated. ^{1}H NMR spectra were measured on a Bruker Avance 500 MHz spectrometer and are recorded as chemical shifts in parts per million downfield from tetramethylsilane ($CDCl_3$ as NMR solvent), multiplicity (s = singlet, d = doublet, q = quartet, m = multiplet), coupling constant (to the nearest 0.5Hz) and assignment, respectively. Coupled GC-MS analysis was performed using a Waters Autospec Ultima mass spectrometer (+EI, 70eV, source temperature 250°C, *m/z* 40-500) coupled to an Agilent 6890 GC fitted with a DB-1 capillary column (J & W Scientific, 50 m x 0.32 mm i.d. x 0.52 μm film thickness) and a cool on-column injector. The oven temperature was programmed to start at 30°C for 5 min, then rise at 5°C/min until 250°C, with a final hold of 10 minutes. The carrier gas was helium. Tentative identification of the EAG-active compound for *C. sordidus* was confirmed by comparison of GC retention time and peak enhancement.

Electrophysiology

Electroantennogram (EAG) recordings were made using Ag-AgCl glass electrodes filled with saline solution [composition as in ref 1 but without the glucose]. The head of an adult female banana weevil, *Cosmopolites sordidus*, was excised and placed within the indifferent electrode and the tips of the antennae were removed before they were inserted into the recording electrode. The signals were passed through a high impedance amplifier (UN-06, Syntech, Hilversum, The Netherlands) and analysed using a customised software package (Syntech). The coupled GC-electrophysiology system, in which the effluent from the GC column is

simultaneously directed to the antennal preparation and the GC detector, has been described previously.[2] Separation of the synthetic isomers of theaspirane and the natural volatile material collected from senesced banana leaf material was achieved on an Agilent 6890 GC equipped with a cool on-column inlet and an FID, using either a DB-1 column (J & W Scientific, 50 m x 0.32 mm i.d. x 0.52 µm film thickness) or a BetaDEX ™ 120 column (Supelco, 30 m x 0.25 mm i.d. x 0.25 µm film thickness). The carrier gas was helium. The oven temperature was maintained at 30°C for 2 minutes and then programmed at 12°/minute to 220°C. The outputs from the EAG amplifier and the FID were monitored simultaneously and analysed using the Syntech software package. A peak was deemed to be electrophysiologically active if it elicited responses on three or more antennal preparations.

Behavioural Assay

A linear, three-chambered olfactometer, comprising of three identical round Perspex arenas and previously described for use with *C. sordidus*[3,4] was used to carry out behavioural bioassays. The olfactometer (Figure S1) was made up of a test chamber in the middle, and two response chambers attached opposite to each other on the sides of the test chamber. Each chamber was provided with a fitting cover, also made of Perspex. The top cover of the test chamber has a vent through which air could be drawn out of the equipment, resulting in a stream of air flowing from the charcoal filters. Each of the three arenas/chambers was about 900 mm of internal diameter, 500 mm height and linked to each other by a 6.5 mm length narrow tube channels also made of Perspex. The channels allowed movement of weevils from one chamber to the other. Black tape was used to bind and cover the entire set up to make it dark and opaque so as to mimic nocturnal environment. The left and right edges of the olfactometer had openings that allowed movement of air into the set up. These vents were

connected with charcoal filters through which the air was filtered before it entered into the chambers. The olfactometer tests were carried out in a black wooden 95 cm x 70 cm x 68 cm (lxbxh) rectangular box that was used to create artificial darkness over the olfactometer to mimic the nocturnal environment similar to that in which the weevils operate. Test weevils used in the bioassay were drawn from within a laboratory-maintained population. Drawn weevils were cultured in a separate container without food (pseudostem). The weevils were starved for 12 hours[4] before they were used in bioassay tests. Bioassays were conducted using groups of ten weevils drawn from the test weevils. The drawn test weevils were placed in the main chamber, and allowed 20 – 30 minutes to respond to and move into stimuli in either response chamber. After this first test period if two (20%) or more weevils failed to decide and respond to either test material, a further 10-15 min was allowed for them to respond. At the end of the permitted time, the number of weevils in each response chamber was recorded.[4] For each experiment, a total of 200 different weevils were used in twenty (20) replicates, and chambers were switched between replicates. Data were analysed using proportionate analyses based on the assumption that ordinarily randomly moving weevils ought to respond to test materials equally if the materials are equally attractive. Students' *t*-tests were used to determine differences between the proportions. Assays comprised of testing (i) senesced banana leaf material versus clean air control (ii) senesced banana leaf volatile organic compounds (VOCs) collected by air entrainment versus a diethyl ether control (iii) synthetic theaspirane (Sigma-Aldrich) solutions in hexane versus a hexane control (iv) theaspirane isomers versus theaspirane isomers (v) senesced banana leaf material versus theaspirane (vi) aggregation pheromone (commercial lure) versus clean air control (vii) aggregation pheromone plus theaspirane versus control (viii) aggregation pheromone plus theaspirane versus aggregation pheromone. For each assay involving extracts, 20 µl of the test solution or control solvent was applied to pieces of filter paper, allowed to dry off for about 5-10 seconds and placed in a pre-

determined test chamber of the olfactometer. Further details of treatments used are described in Tables S1 and S2.

NMR analysis

(*2R,5S*)-theaspirane **1** (500 MHz, $CDCl_3$) ^{1}H-NMR δ: 5.42 (1H, br s, H-9), 4.04 (1H, ddq, *J* = 5.8, 11.4, 5.8 Hz, H-2), 2.12 – 1.96 (4H, m, H-3a, 4a and 8), 1.82 (1H, m, H-4b), 1.72 (3H, br s, 10-Me), 1.69 (1H, dt, *J* = 15.1, 7.6 Hz, H-7a), 1.62 – 1.53 (1H, m, H-3b), 1.31 (1H, dt, *J* = 5.3, 13.0 Hz, H-7b), 1.28 (3H, d, *J* = 6.0 Hz, 2-Me), 0.99 (3H, s, 6-Me), 0.87 (3H, s, 6-Me). ^{13}C-NMR δ: 136.79 (C-10), 123.99 (C-9), 87.57 (C-5), 76.60 (C-2), 37.89 (C-6), 36.19 (C-3), 33.64 (C-7), 31.24 (C-4), 24.07 (6-Me), 22.91 (6-Me and C-8), 21.28 (2-Me), 19.37 (10-Me)

(2*RS*,5*RS*)-theaspirane **1** ^{1}H-NMR δ:.28 (1H, br s, H-9), 4.14 (1H, ddq, *J* = 5.8, 11.4, 5.8 Hz, H-2), 2.10-1.78 (5H, m), 1.73 (3H, br s, 10-Me), 1.56 (1H, ddd, *J* = 6.4, 10.6, 13.4 Hz), 1.47-1.37 (2H, m), 1.29 (3H, d, *J* = 5.9 Hz, 2-Me), 0.97 (3H, s, 6-Me), 0.90 (3H, s, 6-Me).

(2*RS*,5*SR*)-theaspirane **1** ^{1}H-NMR δ: 5.42 (1H, br s, H-9), 4.04 (1H, ddq, *J* = 5.8, 11.5, 5.8 Hz, H-2), 2.12-1.96 (4H, m), 1.82-1.77 (1H, m), 1.72 (3H, br s, 10-Me), 1.69 (1H, dt, *J* = 15.1, 7.6 Hz), 1.62-1.53 (1H, m), 1.31 (1H, dt, *J* = 5.3, 13.0 Hz), 1.28 (3H, d, *J* = 6.0 Hz, 2-Me), 0.99 (3H, s, 6-Me), 0.87 (3H, s, 6-Me).

Assigned ^{1}H-NMR spectrum in $CDCl_3$ at 500 MHz of (*2R,5S*)-theaspirane **1** using the numbering scheme shown in insert

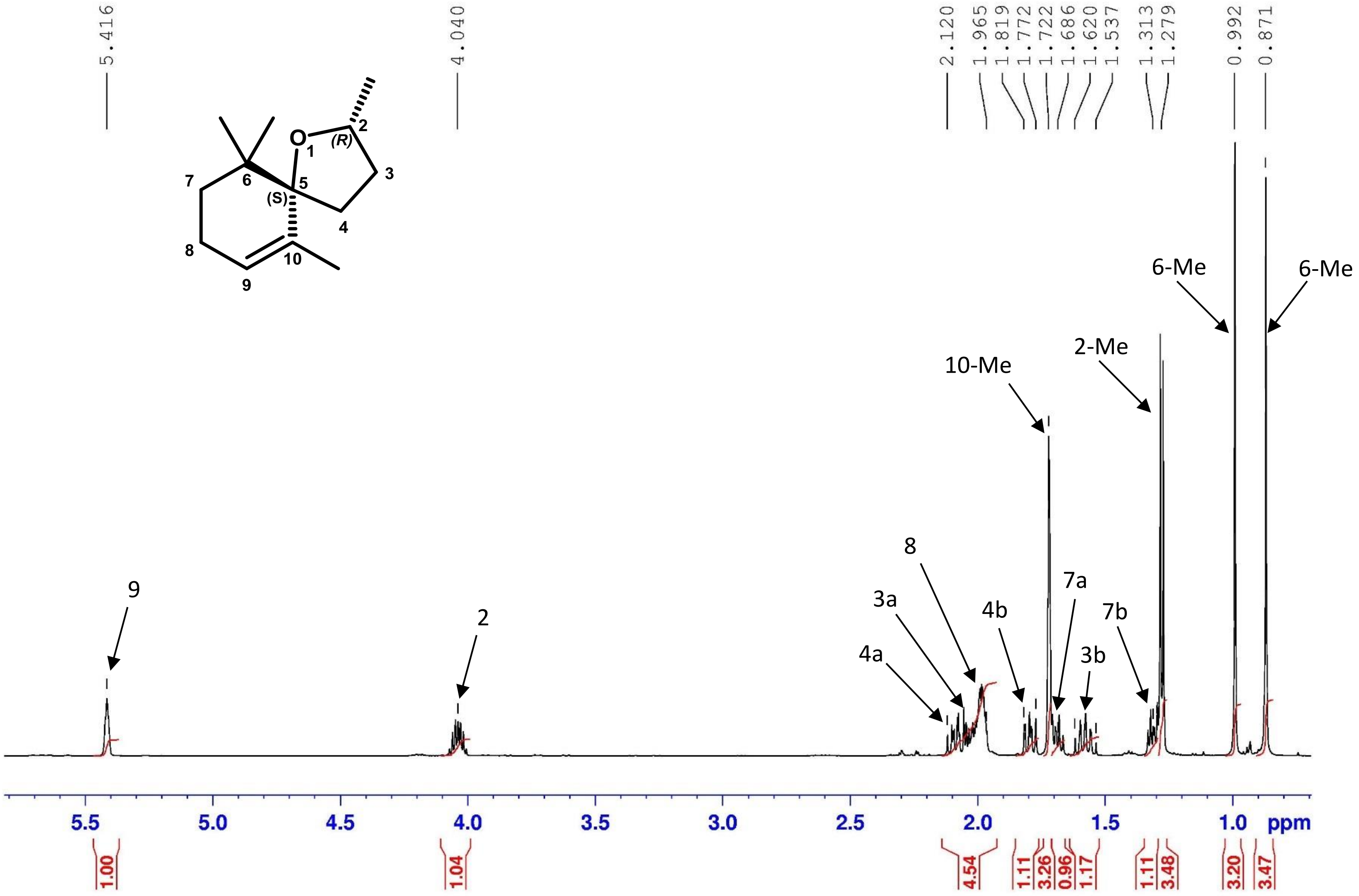

92

A comparison of nOe difference spectra of (*2R,5S*)-theaspirane **1** in $CDCl_3$ irradiated at stated frequencies.

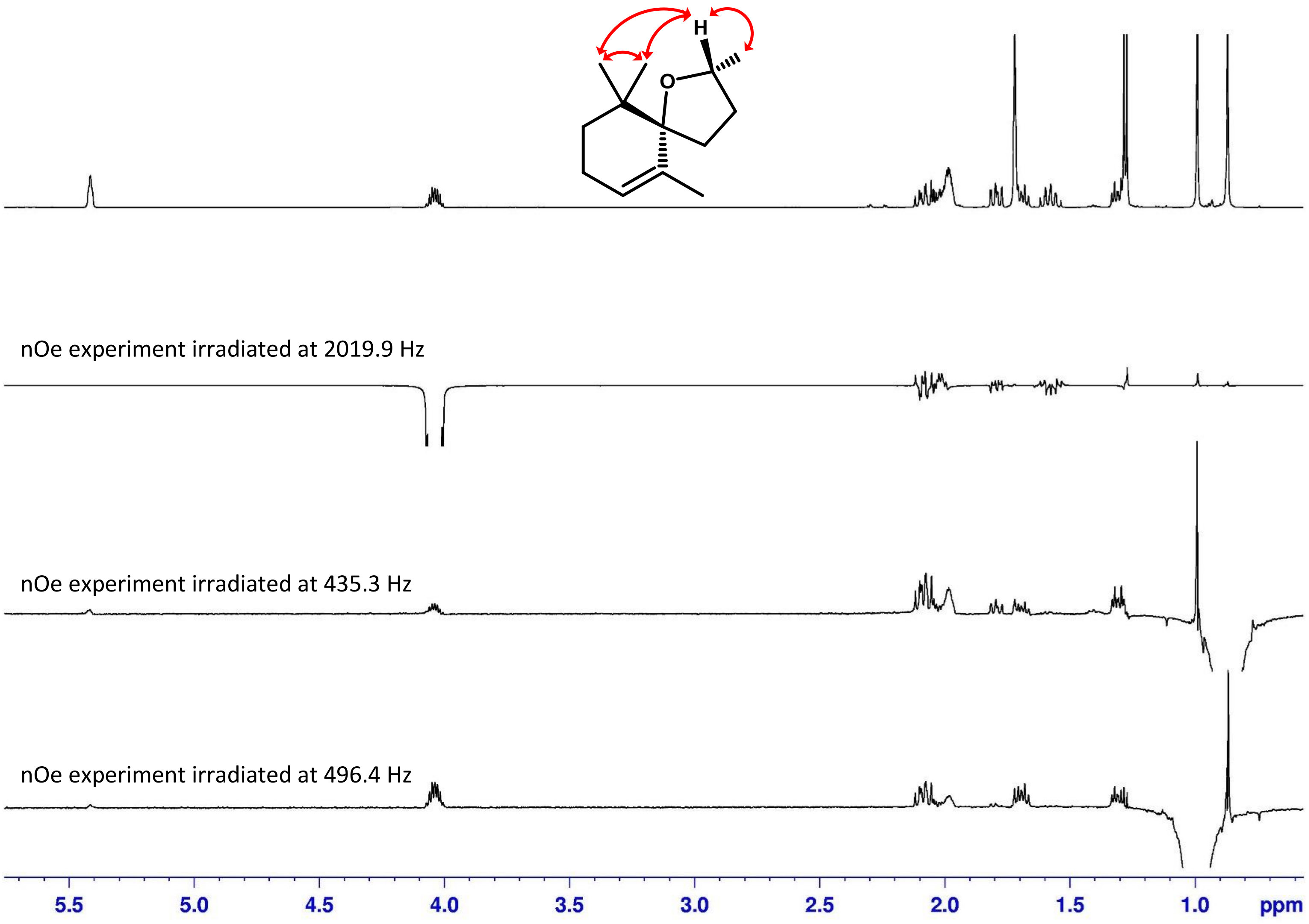

^{13}C-NMR spectrum in $CDCl_3$ at 125 MHz of (*2R,5S*)-theaspirane **1**

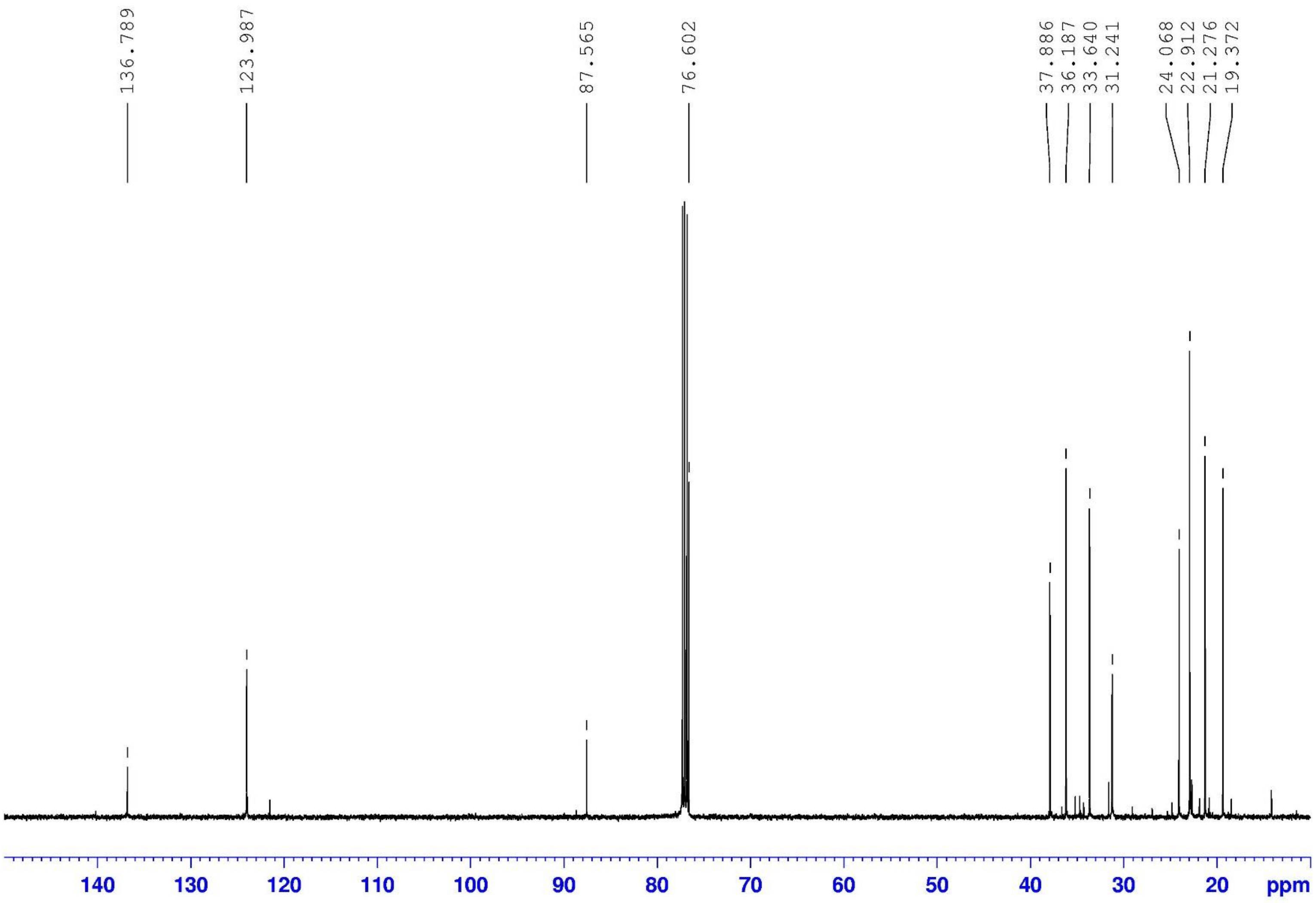

94

^{1}H-COSY spectrum in $CDCl_3$ at 500 MHz of (*2R,5S*)-theaspirane **1**

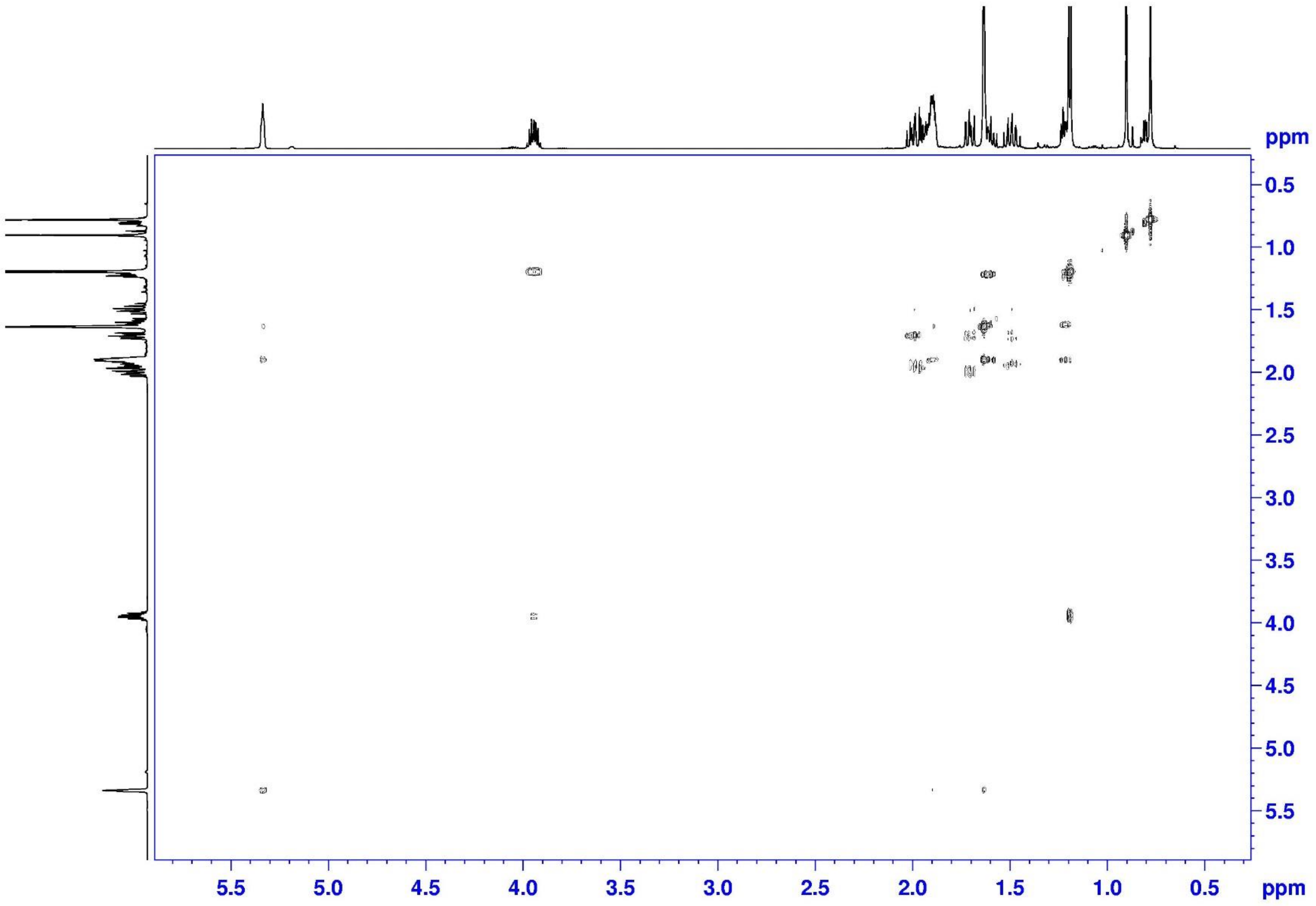

95

HSQC spectrum in $CDCl_3$ of (*2R,5S*)-theaspirane **1**

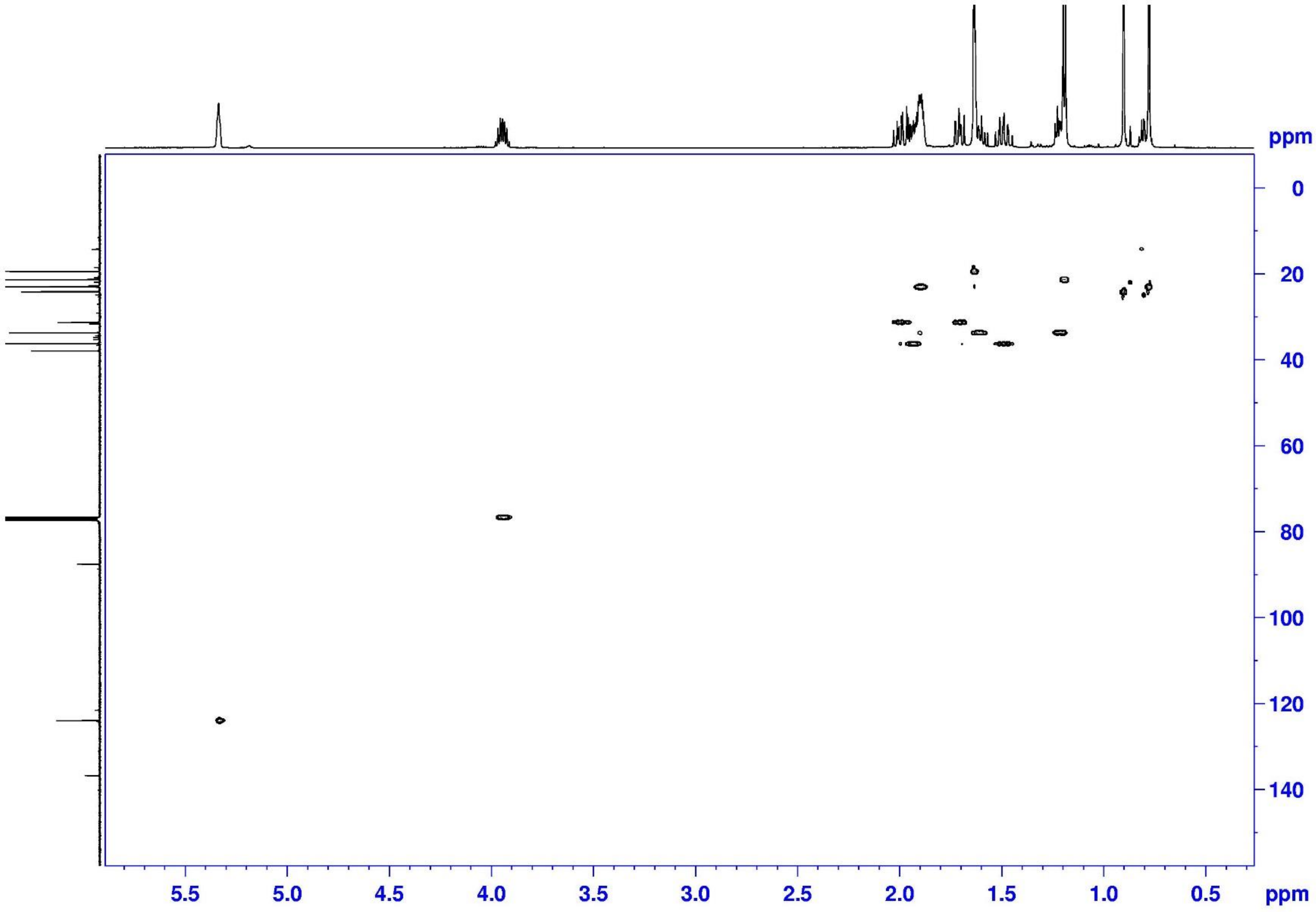

96

HMBC spectrum in $CDCl_3$ of (*2R,5S*)-theaspirane **1**

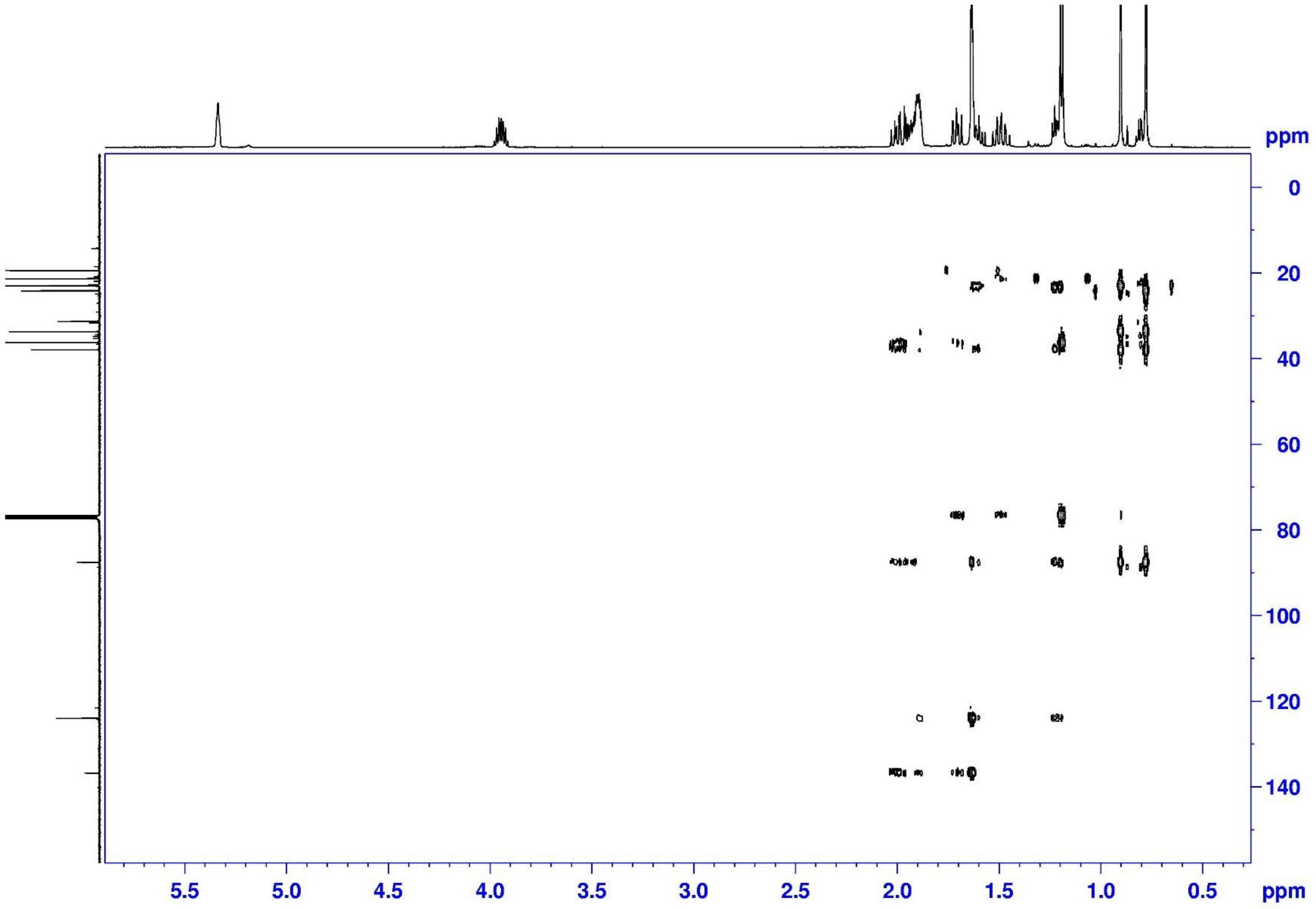

97

Figure S1. Linear, three-chambered olfactometer used to assess behavioural responses of adult banana weevils, *Cosmopolites sordidus*.

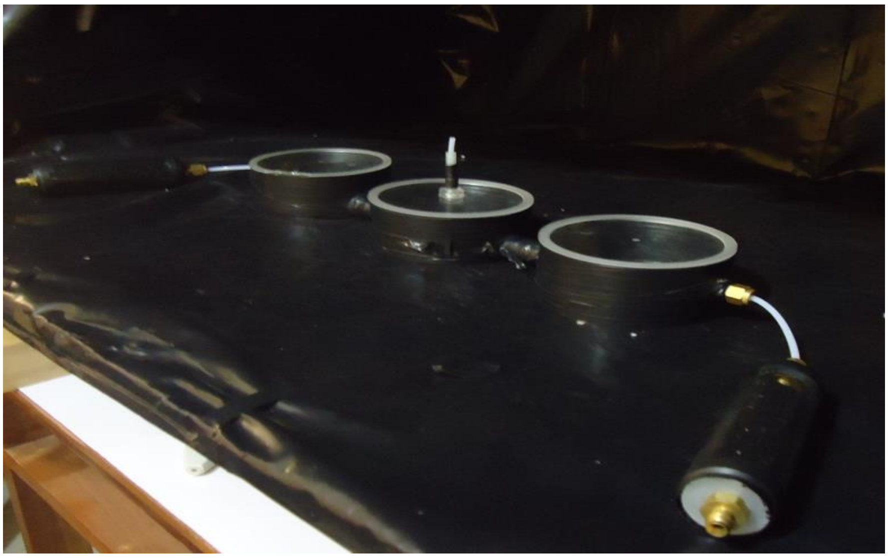

Table S1. Olfactory responses of adult banana weevils, *Cosmopolites sordidus*, to banana leaf material, banana leaf VOCs and theaspirane in a linear olfactometer. Response measured as the mean (± SE) number of weevils found in each chamber of the olfactometer at the end of the experiment. Data were analysed by Students *t*-test. NS = No significant difference between treatments. 20 Replicates were done for each experiment.

**Treatment A**	**Treatment B**	**Mean ± SE**	**Mean ± SE**	***P***
Senesced banana leaf	Clean air	5.55 ± 0.44	3.25 ± 0.42	0.013
Senesced banana leaf VOCs	Diethyl ether	6.30 ± 0.41	3.20 ± 0.42	0.001
Senesced banana leaf VOCs	Senesced banana leaf	4.90 ± 0.51	4.45 ± 0.51	NS
Theaspirane[a]	Hexane	6.10 ± 0.27	3.60 ± 0.28	0.001
Theaspirane[b]	Hexane	5.45 ± 0.30	3.50 ± 0.33	0.017
Theaspirane[c]	Hexane	5.80 ± 0.30	3.30 ± 0.28	0.001
Theaspirane[d]	Hexane	6.00 ± 0.37	3.70 ± 0.37	0.002
Theaspirane[e]	Hexane	5.75 ± 0.41	3.70 ± 0.47	NS
Theaspirane[f]	Hexane	5.75 ± 0.39	3.60 ± 0.41	0.04
Theaspirane[g]	Hexane	4.85 ± 0.36	4.15 ± 0.38	NS
Theaspirane[h]	Hexane	5.45 ± 0.49	3.80 ± 0.46	NS
Theaspirane[i]	Hexane	6.25 ± 0.36	3.35 ± 0.37	0.004
Theaspirane[j]	Hexane	6.20 ± 0.39	2.35 ± 0.43	0.014

[a]Mixture of all 4 isomers of theaspirane at 0.1 μg/ μl total concentration (2 μg total dose)
[b]Mixture of all 4 isomers of theaspirane at 0.1 μg/ μl total concentration (2 μg total dose)
[c]Mixture of all 4 isomers of theaspirane at 0.01 μg/ μl total concentration (0.2 μg total dose)
[d]Mixture of all 4 isomers of theaspirane at 0.001 μg/ μl total concentration (0.02 μg total dose)
[e]Mixture of (2*R*,5*R*)- and (2*S*,5*R*)-theaspirane at 0.1 μg/ μl total concentration (2 μg total dose)
[f]Mixture of (2*R*,5*R*)- and (2*S*,5*R*)-theaspirane at 0.01 μg/ μl total concentration (0.2 μg total dose)
[g]Mixture of (2*R*,5*R*)- and (2*S*,5*R*)-theaspirane at 0.001 μg/ μl total concentration (0.02 μg total dose)
[h]Mixture of (2*S*,5*S*) and (2*R*,5*S*)-theaspirane at 0.1 μg/ μl total concentration (2 μg total dose)
[i]Mixture of (2*S*,5*S*) and (2*R*,5*S*)-theaspirane at 0.01 μg/ μl total concentration (0.2 μg total dose)
[j]Mixture of (2*S*,5*S*) and (2*R*,5*S*)-theaspirane at 0.001 μg/ μl total concentration (0.02 μg total dose)

Table S2. Olfactory responses of adult banana weevils, *Cosmopolites sordidus*, to Cosmolure (commercially available aggregation pheromone) in a linear olfactometer. Response measured as the mean (± SE) number of weevils found in each chamber of the olfactometer at the end of the experiment. Data were analysed by students *t*-test. NS = No significant difference between treatments. 20 Replicates were done for each experiment.

**Treatment A**	**Treatment B**	**Mean ± SE**	**Mean ± SE**	***P***
Cosmolure[a]	Clean air	0.70 ± 0.13	8.60 ± 0.18	<0.001
Cosmolure[b]	Clean air	3.85 ± 0.33	5.55 ± 0.36	0.019
Cosmolure[c]	Clean air	3.90 ± 0.32	5.55 ± 0.32	0.016
Cosmolure[d]	Clean air	5.95 ± 0.27	3.80 ± 0.28	0.004
Theaspirane[e]	Hexane	4.60 ± 0.48	5.00 ± 0.52	NS
Theaspirane[e]	Theaspirane[f]	4.65 ± 0.31	5.20 ± 0.32	NS
Theaspirane[e]	Senesced banana leaf	3.45 ± 0.25	6.30 ± 0.30	<0.001
Cosmolure[d] + Theaspirane[e]	Hexane	5.90 ± 0.30	3.90 ± 0.31	0.014
Cosmolure[d] + Theaspirane[e]	Cosmolure[d]	5.70 ± 0.35	3.95 ± 0.29	0.04

[a]Cosmolure freshly opened
[b]Cosmolure 4 days after opening
[c]Cosmolure 8 days after opening
[d]Cosmolure 14 days after opening
[e]Mixture of (2*S*,5*S*) and (2*R*,5*S*)-theaspirane at 0.0025 µg/ µl total concentration (0.05 µg total dose)
[f]Mixture of all 4 isomers of theaspirane at 0.005 µg/ µl total concentration (0.1 µg total dose)

References

1. S.H.P. Maddrell, *J. Exp. Biol.* 1969, 51, 71.
2. L.J. Wadhams, *Chromatography and Isolation of Insect Hormones and Pheromones.* Plenum Press, New York and London, 1990, 289.
3. H. Braimah, *PhD Thesis*, 1997, University of Reading, UK.
4. H. Braimah and H. F. van Emden, *Bull. Ent. Res.* 1999, 89, 485.
